# Inactivation of SARS-CoV-2 by Simulated Sunlight on Contaminated Surfaces

**DOI:** 10.1128/spectrum.00333-21

**Published:** 2021-07-21

**Authors:** Jérémy Raiteux, Marine Eschlimann, Audrey Marangon, Sophie Rogée, Maylis Dadvisard, Laurent Taysse, Guilhem Larigauderie

**Affiliations:** a DGA CBRN Defence Center, Vert-le-Petit, France; University of Sussex

**Keywords:** emerging disease, SARS-CoV-2, environmental survival, inactivation, interfering substances, stainless steel, sunlight, viruses

## Abstract

We studied the stability of severe acute respiratory syndrome coronavirus 2 (SARS-CoV-2) under different simulated outdoor conditions by changing the temperature (20°C and 35°C), the illuminance (darkness, 10 klx, and 56 klx), and/or the cleanness of the surfaces at 50% relative humidity (RH). In darkness, the loss of viability of the virus on stainless steel is temperature dependent, but this is hidden by the effect of the sunlight from the first minutes of exposure. The virus shows a sensitivity to sunlight proportional to the illuminance intensity of the sunlight. The presence of interfering substances has a moderate effect on virus viability even with an elevated illuminance. Thus, SARS-CoV-2 is rapidly inactivated by simulated sunlight in the presence or absence of high levels of interfering substances at 20°C or 35°C and 50% relative humidity.

**IMPORTANCE** Clinical matrix contains high levels of interfering substances. This study is the first to reveal that the presence of high levels of interfering substances had little impact on the persistence of SARS-CoV-2 on stainless steel following exposure to simulated sunlight. Thus, SARS-CoV-2 should be rapidly inactivated in outdoor environments in the presence or absence of interfering substances. Our results indicate that transmission of SARS-CoV-2 is unlikely to occur through outdoor surfaces, dependent on illuminance intensity. Moreover, most studies are interested in lineage S of SARS-CoV-2. In our experiments, we studied the stability of L-type strains, which comprise the majority of strains isolated from worldwide patients. Nevertheless, the effect of sunlight seems to be similar regardless of the strain studied, suggesting that the greater spread of certain variants is not correlated with better survival in outdoor conditions.

## INTRODUCTION

Coronavirus disease 2019 (COVID-19) is an acute lung injury and acute respiratory distress syndrome that was characterized as a pandemic by World Health Organization (WHO) on 11 March 2020. By 31 March 2021, the disease had spread worldwide, and the WHO reported 128,540,982 confirmed cases of COVID-19, including 2,808,308 deaths (https://covid19.who.int/). COVID-19 is caused by severe acute respiratory syndrome coronavirus 2 (SARS-CoV-2) (1). SARS-CoV-2 belongs to the genus Betacoronavirus and the family Coronaviridae, along with the severe acute respiratory syndrome coronavirus (SARS-CoV) and Middle East respiratory syndrome coronavirus (MERS-CoV) viruses (2). The genome is composed of a single-stranded positive-sense RNA. SARS-CoV-2 is the third lethal human coronavirus that has emerged since 2003, after SARS and MERS coronaviruses (3). Nevertheless, SARS-CoV-2 induces a relative low mortality rate (3.4%) in comparison to those of SARS-CoV (9.6%) and MERS-CoV (34.4%) (4); however, SARS-CoV-2 is much more contagious (5). SARS-CoV-2 can be transmitted either by direct person-to-person contact or by indirect routes such as contaminated inanimate (6, 7).

Under laboratory conditions, it has been shown that coronaviruses are stable on inanimate surfaces (8). Indeed, investigations have demonstrated that SARS-CoV-2 could stay viable from 3 h (copper) to 6 days (plastic and stainless steel) (9, 10). Moreover, even if the detection of viral RNA is not necessarily correlated with infectious particles, viral RNA has been detected on different surface samples surrounding hospitalized COVID-19 patients (11–13) and in community settings such as airports and schools. Thus, contaminated environmental surfaces could play an important role in SARS-CoV-2 transmission.

The evaluation of SARS-CoV-2 stability under both indoor and outdoor conditions is necessary to understand the virus transmission in order to limit the spread of the virus. Three major factors might affect virus viability: temperature, humidity and solar radiation. Radiation emitted by sunlight is the primary cause of virus inactivation in the atmosphere (14). It has been known for a long time that radiation is absorbed by the nucleic acids of the virus and induces chemical modifications (15). Among these types of radiation, UVC (wavelength from 100 nm to 280 nm) light effectively inactivates coronaviruses (16). UV radiations with a wavelength of at least 290 nm (UVB and UVA) reach the surface of the Earth, but a very low level of UVC reaches the surface of the Earth due to absorption by the atmosphere (17). Thus, our study focused on UVA and UVB. Using a model estimating solar inactivation of the virus, it was previously calculated that 90% of SARS-CoV-2 is inactivated after 11 to 34 min of midday sunlight exposure in North America in summer (18). This exposure is similar during summer in France. A recent study shows that 90% of the SARS-CoV-2 suspended in simulated saliva or culture medium and dried on stainless steel is inactivated every 6.8 min and every 14.3 min, respectively, when the virus is exposed to simulated sunlight representative of the summer solstice. This study was the first investigation analyzing the effect of simulated sunlight on inactivation of SARS-CoV-2 dried on inanimate surfaces. Nevertheless, the antiviral activity of simulated sunlight was only evaluated at a single temperature (20°C ± 4°C), a relative humidity (RH) of 19% ± 5% (a very dry climate; in most climates, outside relative humidity is higher than 20%), and with a low level of interfering substances (19).

The aim of our present study is to evaluate the impact of multiple luminescence intensities of simulated sunlight and different temperature levels under controlled humidity (relative humidity of 50%), reflecting the autumn and summer seasons in France, on SARS-CoV-2 dried on stainless steel. This inanimate surface represents common sources of fomite transmission (e.g., door knobs and subway grab bars). Clinical matrices such as saliva mucus, stool samples, or blood, contain a high level of substances that interfere with viral survival. Therefore, with a high level of interfering substances composed of 1% bovine serum albumin (BSA) and 1% yeast extract (following the NF EN 14675 standard, which describes a suspension test to evaluate the virucidal activity of chemical disinfectants intended for use in the veterinary area [20]).

## RESULTS

### In darkness, the viability of SARS-CoV-2 depends on temperature.

**FIG 1:**
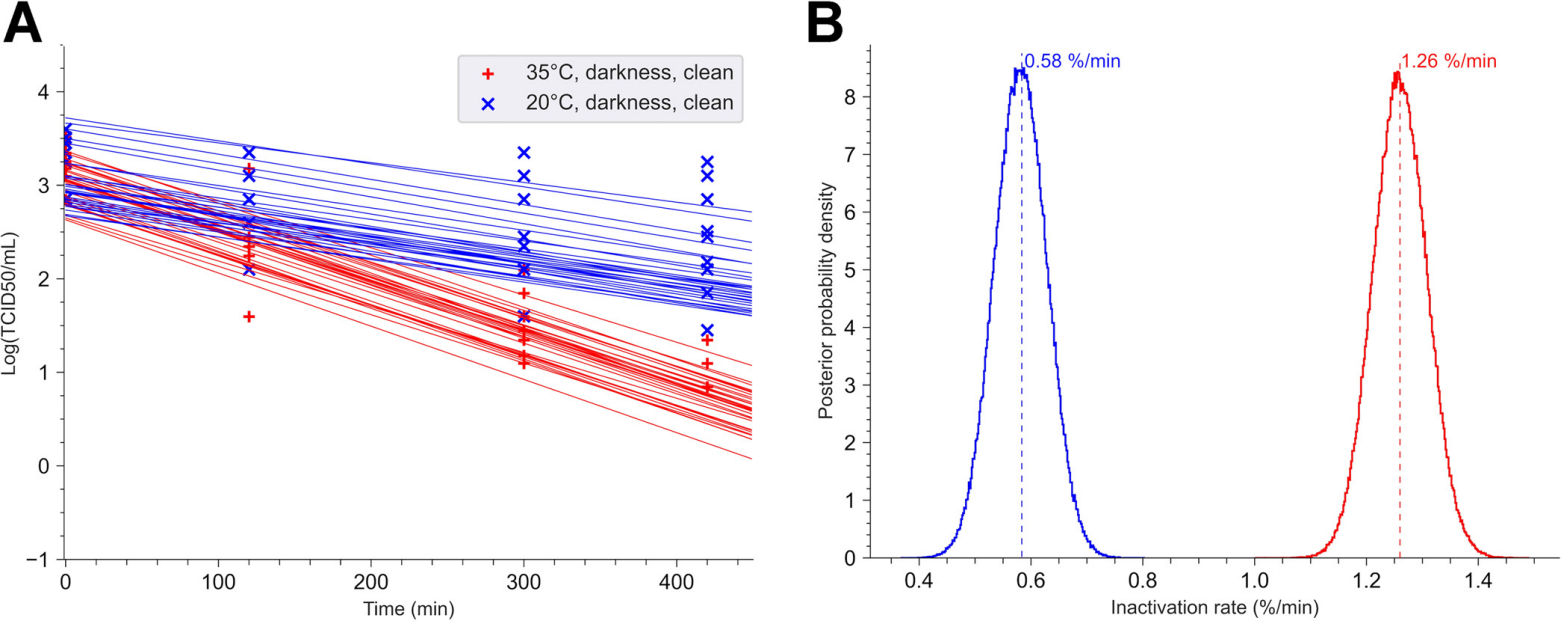
Impact of temperature on SARS-CoV-2 stability in the darkness. In small stainless-steel coupons, 10 μl of a suspension of SARS-CoV-2 at 3.43 × 10^6^ 50% tissue culture infectious dose (TCID_50_)/ml was deposited and dried at room temperature for 45 min. Then, the coupons with dried viral supernatant were exposed in a chamber at 35°C or 20°C in darkness. The relative humidity was regulated and maintained at 50% (± 5%). For each point of the kinetics analysis, three coupons were taken. Each experiment was independent and was repeated three times. (A) Bayesian inference was conducted on these nine (3 × 3) data sets. For each condition, we plotted 30 random lines from the Bayesian inference. Each point represents the log_10_ of tissue culture infectious dose estimated by the Reed-Muench method. (B) Posterior probability densities for decay rate estimated by the No U-turn sampler algorithm, under our Bayesian model, are represented for each condition. The 35°C-darkness-clean surface condition is indicated in red (mean value, 1.26% per min; 94% highest density interval [HDI], 1.15 to 1.37) and the 20°C-darkness-clean surface condition in blue (mean value, 0.58% per min; 94% HDI, 0.47 to 0.7).

We first investigated the impact of temperature on the viability of the virus in darkness (Fig. 1) on clean surfaces at 50% humidity.

The kinetics of virus inactivation were performed during 7 h in darkness at both 20°C and 35°C. These temperatures were chosen to simulate the environmental conditions in summer and autumn in France, based on meteorological data collected at Vert-Le-Petit (France). At time zero (T0), which represented the concentration of infectious viral particles recovered in the coupon following 45 min of drying, we quantified only 1.5 × 10^3^ 50% tissue culture infective dose (TCID_50_), whereas a concentration of 3.43 × 10^4^ TCID_50_ was deposited. This reduction was probably correlated with desiccation of viral suspension (21). The decay rate in min^−1^*k* was defined as percent of decay per min (100*k*) (see supplemental material). At 35°C, according to our results, the inactivation of the virus was significantly faster than that at 20°C (*p*-direction = 0) (Fig. 1A). After 7 h of exposure, there was 10 times more active virus at 20°C than at 35°C. Indeed, the percent decay rate per minute changed from 1.26% per min at 35°C to 0.58% per min at 20°C (Fig. 1B).

### Illuminance impacted the viability of SARS-CoV-2 instantly, compared to the less significant effect of temperature.

**FIG 2:**
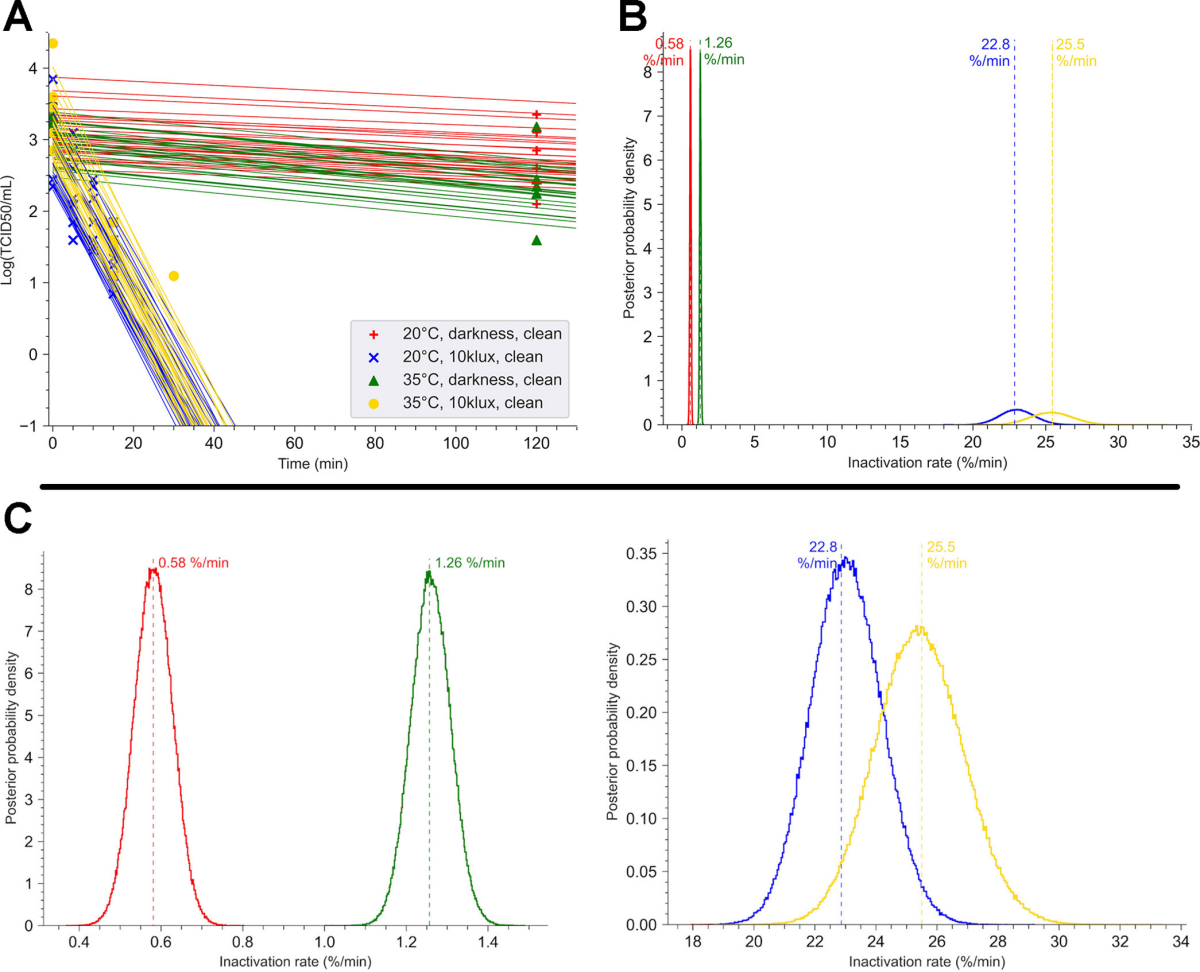
Impact of illuminance and temperature on SARS-CoV-2 stability. Twelve coupons loaded with 10 μl of a dried suspension of SARS-CoV-2 at 3.43 × 10^6^ TCID_50_/ml were exposed in a chamber in darkness at 35°C or 20°C. The decay of SARS-CoV-2 was calculated from the titer of virus recovered in the coupon following exposure. Experiments were performed in darkness or at an illuminance of 10 klx. Relative humidity was maintained at 50%. (A) For each point of the kinetics analysis, three coupons were taken. The time zero (T0) point was defined as the concentration of infectious viral particles recovered in three different coupons following 45 min of drying. Each experiment was independent and was repeated three times. Bayesian inference was conducted on these nine (3 × 3) data sets. For each condition, we plotted 30 random lines from the Bayesian inference. Points represent the virus titer (log[TCID_50_]) estimated by Reed-Muench method. (B) Posterior probability densities for decay rate estimated by the No U-turn sampler algorithm, under our Bayesian model, are represented for each condition. The 20°C-darkness-clean surface condition is indicated in red, and the 35°C-darkness-clean surface condition is indicated in green. The 20°C-10 klx-clean surface condition is represented in blue, and the 35°C-10 klx-clean surface condition in gold. (C) Detail of the estimated posterior probability densities for decay rate. (Left) Darkness conditions at 20°C in red (mean value, 0.58% per min; 94% HDI, 0.48 to 0.72) and at 35°C in green (mean value, 1.26% per min; 94% HDI, 1.12 to 1.38). (Right) Estimated posterior probability densities for decay rate following exposure at 10 klx with either 20°C in gold (mean value, 22.8% per min; 94% HDI, 20 to 26.2) or 35°C in purple (mean value, 25.5% per min; 94% HDI, 21.7 to 29.5).

Next, we focused our study on the impact of illuminance (the density of luminous flux on a delimited surface) intensity on the virus survival. A solar simulator with a xenon lamp was used. At 20°C and 35°C and 50% humidity, the virus was exposed to an illuminance of 10 klx (equivalent to an irradiance of 79 W/m^2^), comparable to cloudy weather. Figure 2A presents the effect of sunlight compared with the effect of temperature. At both temperatures, the virus was not detectable at the end of 20 min for an illuminance of 10 klx, whereas infectious viral particles were still measurable at 400 min in darkness (577.1 TCID_50_ at 20°C and 28.6 TCID_50_ at 35°C). According to the No U-Turn sampler algorithm, under our Bayesian model for each condition, the decay rates for an illuminance of a 10-klx exposure were significantly faster (22.8% per min at 20°C and 25.5% per min at 35°C) than those obtained in darkness (0.58% per min at 20°C and 1.26% per min at 35°C) (*p*-direction = 0 for each temperature for darkness versus 10 klx) (Fig. 2B and C).

### The virus shows sensitivity to sunlight proportional to illuminance intensity.

**FIG 3:**
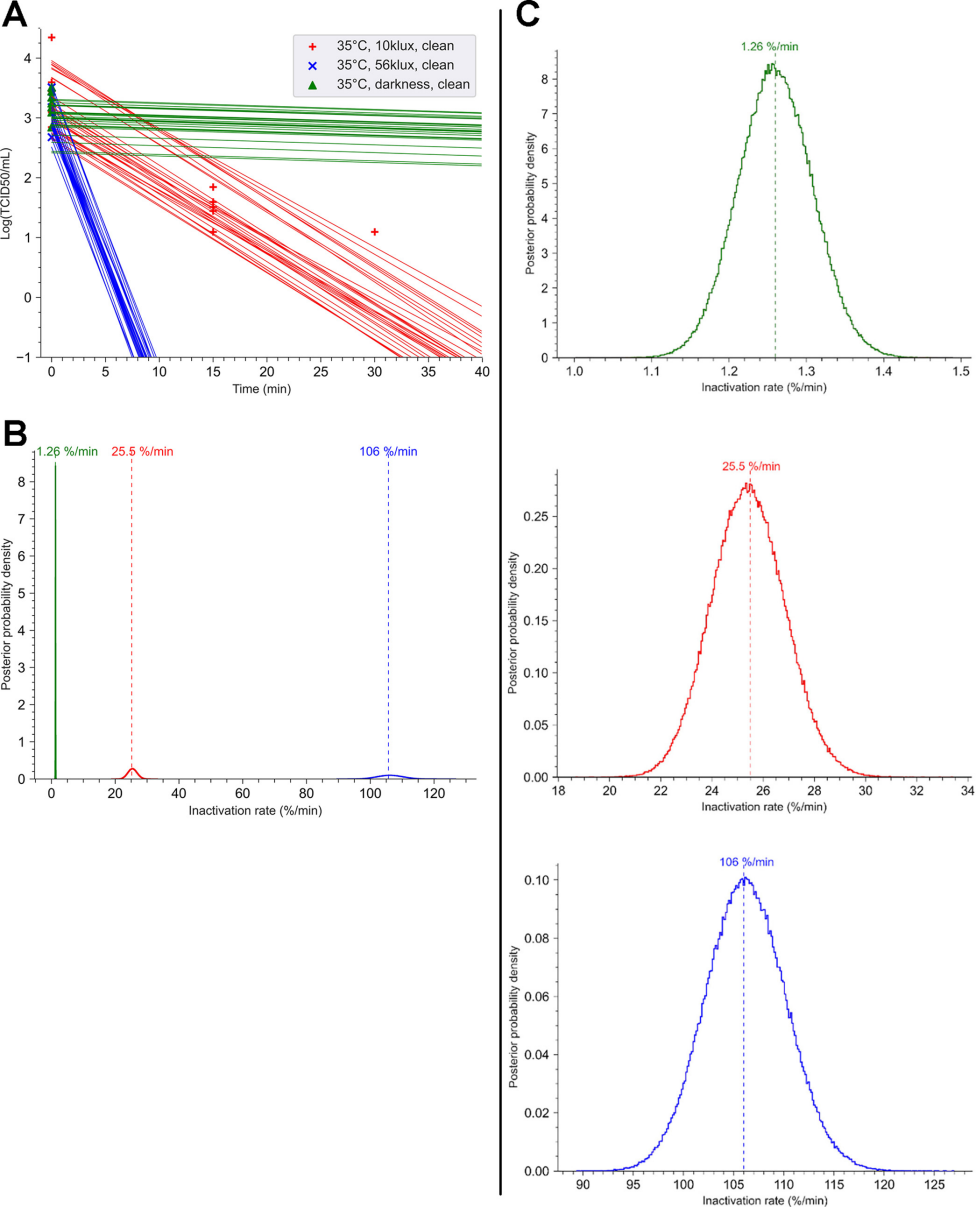
Decay of SARS-CoV-2 is dependent of illuminance at 35°C. A suspension of SARS-CoV-2 (10 μl at 3.43 × 10^6^ TCID_50_/ml) was deposited in small stainless-steel coupons. Coupons were dried at room temperature (RT) for 45 min and then exposed in a chamber at 35°C with an illuminance of 10 or 56 klx or in the darkness. The relative humidity was adjusted to 50%. (A) For each point of the kinetics analysis, three coupons were taken. Each experiment was independent and was repeated three times. Bayesian inference was conducted on these nine (3 × 3) data sets. For each condition, we plotted 30 random lines from the Bayesian inference. Points represent the virus titer (log[TCID_50_]) estimated by the Reed-Muench method. Three coupons were taken at time zero (T0). (B) Posterior probability densities for decay rate estimated by the No U-turn sampler algorithm, under our Bayesian model, are represented for each condition. The 35°C-10 klx-clean surface condition is shown in red, the 35°C-56 klx-clean surface condition is in blue, and the 35°C-darkness-clean surface condition is in green. (C) Detail of the posterior probability densities for estimated decay rate. (Left) The 35°C-darkness-clean surface condition (mean value, 1.26% per min; 94% HDI, 1.14 to 1.38). (Middle) The 35°C-10 klx-clean surface condition (mean value, 25.5% per min; 94% HDI, 21.7 to 28.4). (Right) Posterior probability densities for decay rate following exposure at 56 klx at 35°C, shown in gold (mean value, 106% per min; 94% HDI, 96 to 117).

Then, we investigated the impact of various illuminance intensities on virus viability at 35°C and 50% humidity. The following three illuminance conditions were studied: darkness, 10 klx, and 56 klx (equivalent to an irradiance of 442 W/m^2^). The inactivation kinetics and regression linear fit of SARS-CoV-2 recovered from stainless steel coupon after different experiment conditions as a function of the illuminance intensity are shown in Fig. 3A. At 35°C, infectious virus was still detectable up to 20 min at 10 klx. For an illuminance of 56 klx, infectious virus was no longer detectable after 5 min of exposure. Ninety percent of viral load was lost every 9 min and 12 s at an illuminance of 10 klx. At 56 klx, this time was reduced to 2 min and 12 s. These results indicated that the decay rate increased from 25.5% to 106% per minute for illuminance from 10 klx to 56 klx (Fig. 3B and C). Under lighting conditions, the rates of inactivation of the virus were significantly faster than those obtained in darkness (*p*-direction = 0).

### Interfering substances had a moderate impact on the virus viability under elevated illuminance.

To study the effect of the cleanness of surfaces, we simulated a high level of proteins by adding interfering substances (BSA and yeast extract) to the virus suspension.

**FIG 4:**
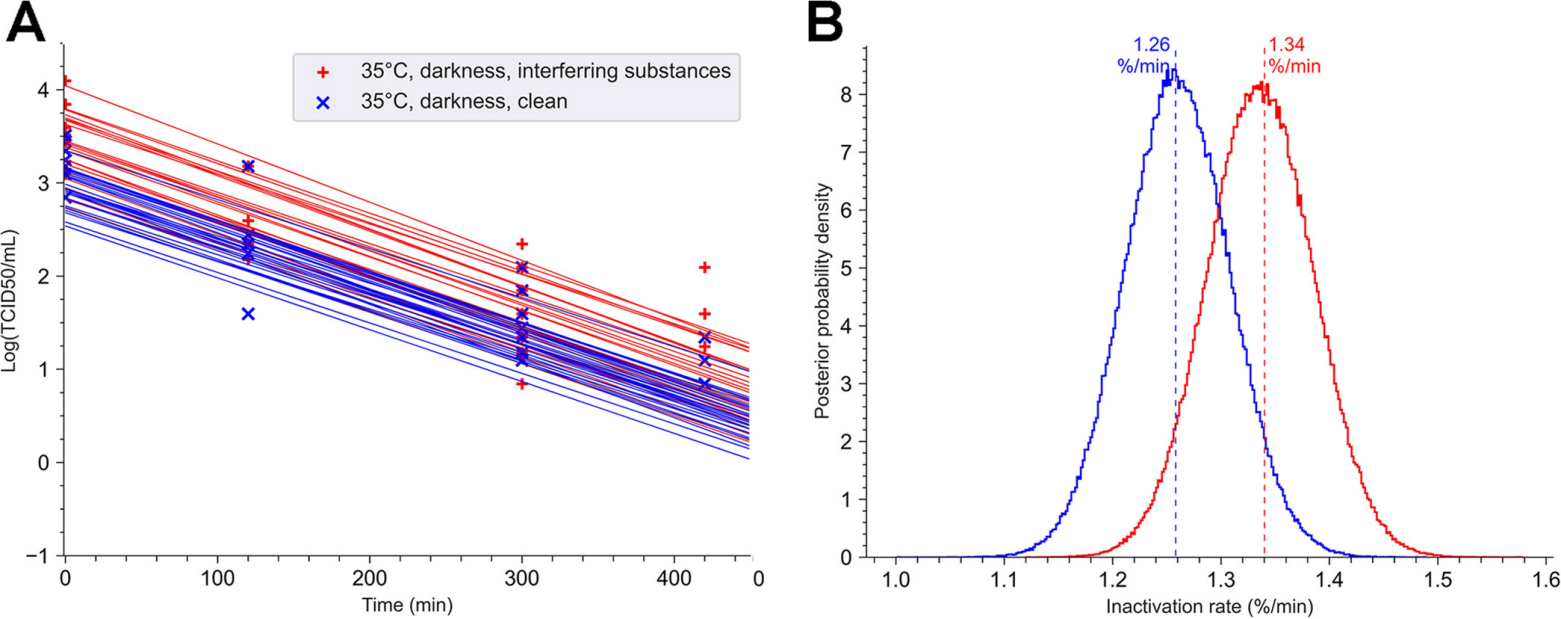
Role of interfering substances in the stability of SARS-CoV-2 in darkness. Twelve coupons loaded with 10 μl of a suspension of SARS-CoV-2 at 3.43 × 10^6^ TCID_50_/ml, with or without interfering substances (1% BSA and 1% yeast extract). Droplets were allowed to dry on coupons for 45 min. The mean of the tissue culture infectious dose after drying was calculated and corresponded at T0. The coupons were exposed in a chamber at 35°C in the darkness. The relative humidity was regulated and was maintained at 50%. (A) For each point of the kinetics analysis, three coupons were taken. T0 was defined as the concentration of infectious viral particles recovered in three different coupons following 45 min of drying. Each experiment was independent and was repeated three times. Bayesian inference was conducted on these nine (3 × 3) data sets. For each condition, we plotted 30 random lines from the Bayesian inference. Points represent the virus titer (log[TCID_50_]) estimated by the Reed-Muench method. (B) Posterior probability densities for the decay rate estimated by the No U-turn sampler algorithm, under our Bayesian model, are represented for each condition. The 35°C-darkness-with inferring substances condition is indicated in red (mean value, 1.34% per min; 94% HDI, 1.22 to 1.48) and the 35°C-darkness-clean surface condition (mean value, 1.28% per min; 94% HDI, 1.14 to 1.38) is in blue.

**FIG 5:**
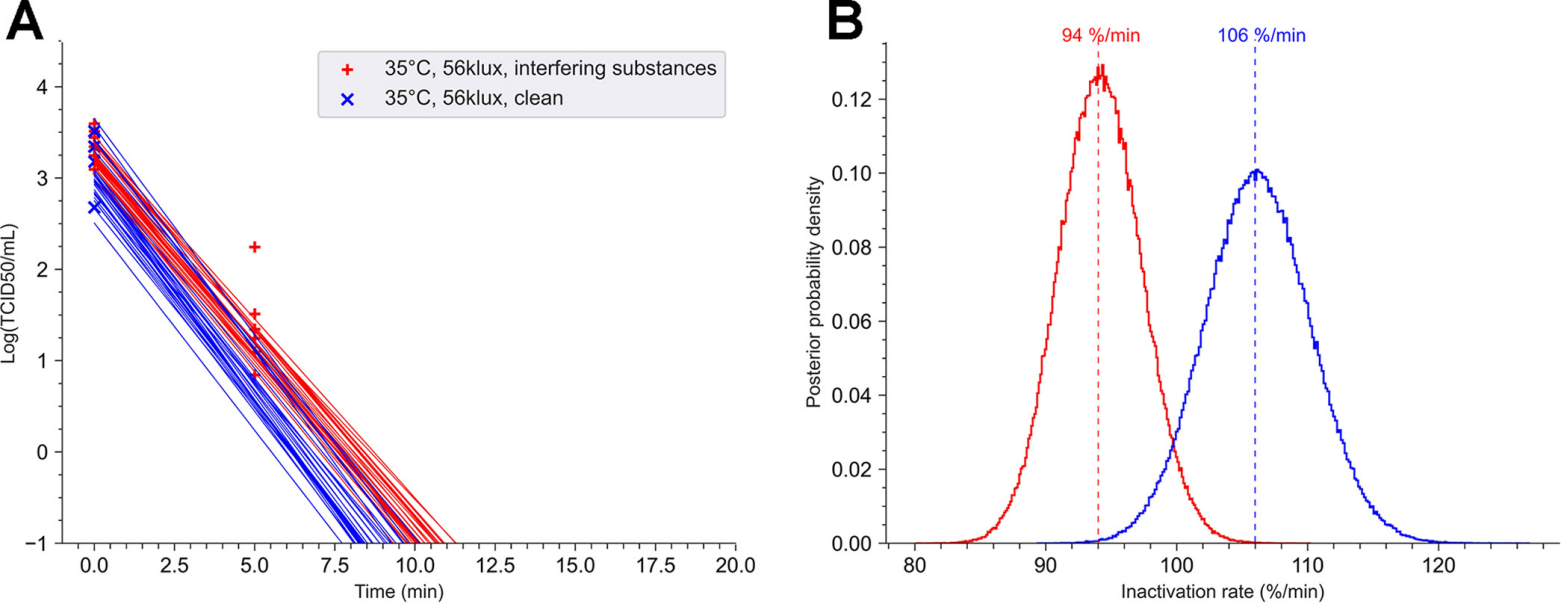
Decay kinetics of SARS-CoV-2 as a function of interfering substances at 35°C and 56 klx. A suspension of SARS-CoV-2 (10 μl at 3.43 × 10^6^ TCID_50_/ml) in the presence or absence of interfering substances was deposited in small stainless-steel coupons. Coupons were dried at RT for 45 min and were then exposed in a chamber at 35°C with an illuminance of 56 klx. The relative humidity was regulated and was maintained at 50%. (A) For each point of the kinetics analysis, three coupons were taken. Each experiment was independent and repeated three times. Bayesian inference was conducted on these nine (3 × 3) data sets. For each condition, we plotted 30 random lines from the Bayesian inference. Points represent the virus titer (log[TCID_50_]) estimated by the Reed-Muench method. (B) Posterior probability densities for the decay rate were estimated by No U-Turn sampler algorithm, under our Bayesian model, for each condition. The 35°C-56 klx-with inferring substances condition (mean value, 94% per min; 94% HDI, 86 to 103) is represented in red, and without interfering substances in blue (mean value, 106% per min; 94% HDI, 96 to 117).

The impact of interfering substances on the persistence of SARS-CoV-2 at 35°C (Fig. 4A) in darkness was first analyzed. At this temperature, no significant difference was observed. The decay rate was approximatively 1.3% per minute under clean conditions or with interfering substances (Fig. 4B). Those results show that interfering substances did not affect virus viability at 35°C in the darkness. Based on this result, the effect of the interfering substances was investigated at 35°C and 50% humidity for an illuminance of 56 klx (Fig. 5). The virus was no longer quantifiable after 5 min on clean surfaces, while with interfering substances, the virus was still detectable at this period of exposure. However, the virus was no longer detectable in less than 10 min, even when in contact with interfering substances (Fig. 5A). These results indicate that the impact of interfering substances on the survival of SARS-CoV-2 in comparison with that under clean conditions (decay rate of 94% per minute versus 106% per minute) is low but statistically significant (*p*-direction, <0.02) (Fig. 5B).

## DISCUSSION

Our study confirmed that in darkness, the decay rate of SARS-CoV-2 on stainless steel is dependent on temperature (22, 23). However, we observed different half-life values of SARS-CoV-2 on stainless steel (119 min at 20°C and 53 min at 35°C at 50% RH) in comparison to the mean of half-life values reported in previous articles (9, 10, 22–24). The varied persistence of SARS-CoV-2 estimated in these different articles (from 2.5 h to 11.54 h) could be explained by different experimental conditions, including temperature (from 19°C to 40°C), relative humidity (20% to 70%), the initial load, or the drying exposure time. Indeed, it has been shown that these different factors could have an impact on the stability of SARS-CoV-2. Compared with the half-lives of other respiratory viruses, such as influenza A (H1N1), SARS-CoV, and MERS-CoV, the half-life of SARS-CoV-2 is similar to those of SARS-CoV and MERS-CoV (10, 25), but the virus appears to be more stable than H1N1 (30 min at 22°C and 50 to 60% RH) (26).

Currently, very few data are available on the impact of sunlight on the survival of respiratory viruses on inanimate surfaces. Sunlight is composed of UVA, UVB, and UVC radiation. Many investigations have shown the efficacy of UVC against SARS-CoV-2 (16). Nevertheless, outdoors, a very low level of UVC reaches the Earth’s surface. Our investigation revealed that simulated sunlight with UVA and UVB radiations rapidly inactivates SARS-CoV-2. This inactivation was independent of temperature under our experimental conditions. We investigated two temperatures for comparisons of illuminance, 20°C and 35°C, which induced a lower decay rate. Following exposure at 10 klx, 90% of SARS-CoV-2 virus was inactivated within 10 min regardless of the temperature tested. We suggest that the impact of illuminance on SARS-CoV-2 inactivation is so rapid that it hides the effect of the temperature, which is observable at longer kinetic times in the dark. It was previously shown that in indoor conditions, the persistence of SARS-CoV-2 is longer at 4°C than at 21°C (9). It would be interesting to investigate the impact of illuminance at lower temperatures, such as 4°C to mimic winter conditions. However, simulated sunlight induces a heating of the chamber. Maintaining a temperature at 4°C is a challenge and requires tool optimization. At 35°C, we also observed that the decay rate is completely dependent on illuminance intensity. Ninety percent of infectious virus was degraded within 9 min at 10 klx and in 2 min and 17 s at 56 klx. Thus, our results corroborated the data obtained by Ratnesar-Shumate et al. under similar experimental conditions (19). Our results show a decay rate several time faster than that predicted by the theory based on UVB radiation (18, 27). The spectrum of the xenon lamp contained both UVA and UVB radiation. The virucidal action observed in our data is potentially linked to the combined effect of UVA and UVB, in which the UVA portion is less efficient than UVB (28, 29).

The spread of SARS-CoV-2 might be associated with socioeconomic factors (poor sanitary and medical conditions) (30, 31), density of population (32), and the concentration of fine atmospheric particulates (33). Nevertheless, a correlation between illuminance or UV light and lower COVID-19 growth has been reported (30, 34), suggesting that COVID-19 might be seasonal. Our data showed that the decay rate of SARS-CoV-2 is correlated with illuminance intensity. The severity of COVID-19 could be also correlated with hypovitaminosis D. The UV in sunlight increases vitamin D synthesis after exposure (35, 36). Thus, sunlight has a virucidal activity but could also participate somewhat in the prevention of lethal cases of COVID-19 via increasing synthesis of vitamin D.

Ratnesar-Shumate et al. (19) used the SARS-CoV-2 USA-WA1/2020 strain, which belongs to the S lineage and represents 30% of strains isolated from patients worldwide (37). In our investigation, we used the BetaCov/France/IDF-0372/2020 strain. This strain differs by only two single-nucleotide polymorphisms (SNPs) from the Wuhan HU-1 strain and belongs to the same lineage (L lineage). L-type strains are more prevalent and tend to be more aggressive and contagious than S-lineage strains (37). After alignment using the progressiveMauve aligner (38), the comparison of these two sequences shows five SNPs, including one nonsynonymous mutation in the spike gene (nucleotide G at position 22661 in the Wuhan-HU-1 and USA-WA1 strains versus T in BetaCov/France/IDF-0372/2020) (see Table S1 in the supplemental material). However, these mutations did not influence the persistence of SARS-CoV-2 after simulated sunlight exposure. We hypothesized that simulated sunlight should have similar effects on different strains of SARS-CoV-2.

As a respiratory virus, SARS-CoV-2 is mainly spread by airborne transmission, and possibly by fomites (39). Environmental surfaces may be contaminated by droplets expelled by infected persons speaking, coughing, or sneezing or by indirect transfer from hands contaminated with excreted virus (39, 40). Some studies have evaluated the stability of SARS-CoV-2 suspended in culture medium or simulated saliva. These data revealed that the simulated saliva had no or a moderately negative effect on the viability of SARS-CoV-2. These effects seem to be correlated with the temperature and the sunlight intensity exposure (19, 41). Unlike mucus and saliva, interfering substances mimicking high protein levels induced a better persistence of coronavirus (42). Pastorino et al. demonstrated that SARS-CoV-2 stability was significantly preserved in the presence of BSA, with a half-life of more than 96 h versus 2.5 h without BSA (24). In darkness, we did not observe an effect of interfering substances on the persistence of SARS-CoV-2 dried on stainless steel. Nevertheless, our kinetics analysis is short, and Pastorino et al. (24) showed an impact of BSA after more than 8 h of incubation. In the presence of simulated sunlight with an illuminance of 56 klx, we observed a moderate impact of the interfering substances on the survival of SARS-CoV-2 dried on stainless steel. Indeed, the decay rate was estimated as 94% per minute in the presence of interfering substances versus 106% per minute under clean conditions. The proteins contained in the interfering substances could act as a light protective barrier for the viral particles and increase virus viability. Nevertheless, the aromatic residues of proteins can capture UV radiations (from ∼250 to 298 nm) and can induce structural changes such as denaturation and protein fragmentation, particularly in dehydrated systems (15). Therefore, following sunlight exposure at 56 klx, interfering substances are probably rapidly degraded. That is why SARS-CoV-2 inactivation remains very rapid, with a half-life of less than 1 min.

Our study confirms that simulated sunlight rapidly inactivates SARS-CoV-2 at temperatures ranging from 20°C to 35°C. A high level of interfering substances had a very slight impact on the persistence of the virus after exposure to the sun. Moreover, the decay rate of SARS-CoV-2 is correlated with illuminance intensity of the sunlight. Our data also suggest that sunlight could be preventive of SARS-CoV-2 spread.

## MATERIALS AND METHODS

### Cell culture.

Vero E6 cells were used to titrate infectious particles of SARS-CoV-2. These cells were cultured at 37°C, under 5% CO_2_ in Dulbecco’s modified Eagle medium (DMEM) with GlutaMax (catalog no. 31966-021; Gibco), supplemented with 10% heat-inactivated fetal bovine serum (FBS) (catalog no. 10270-106; Gibco) and 1× antibiotic-antimycotic solution (catalog no. MS005Q1; Biowest).

### Viral production.

The strain BetaCov/France/IDF-0372/2020 was obtained from the European Virus Archive catalog (EVA-GLOBAL H2020 project). This strain was isolated at the Pasteur Institute in Paris from a French patient returned from China. Virus production and experiments were performed in confined laboratories according to French biosafety guidelines. A viral suspension of 3.43 × 10^6^ TCID_50_/ml was used for the different experiments. Confluent monolayers of Vero E6 cells (ATCC CRL-1586) were infected with a multiplicity of infection (MOI) of 0.01. The cells were then incubated at 37°C at 5% CO_2_. At 48 h postinfection, cells and supernatant were collected and clarified by centrifugation at 4,000 × *g* for 5 min and aliquoted. Aliquots were then stored at −80°C until use. The concentration of infectious virus was determined by microtitration according to the protocol presented below.

### Virus titration.

The concentration of viral production and viral particles harvested from surfaces was determined as follows. A titration by limit dilution was performed with serial 1:10 dilution for each coupon or viral production in DMEM medium with GlutaMax supplemented with 2% heat-inactivated fetal bovine serum (FBS) and 1× antibiotic-antimycotic solution. Undiluted viral sample and each dilution were deposited in six wells of the 96-well plate, at a rate of 200 μl per well, and were then incubated at 37°C at 5% CO_2_. To quantify the infectious particles of SARS-CoV-2, the TCID_50_ Reed-Muench calculation method was used (43).

### Preparation of coupons and exposure chamber.

The inert surface used for the experiments was a 9-mm circular stainless-steel coupon. This material was chosen because of it is representative of various everyday items, such as cutlery or door handles. Moreover, this inert material did not create a bias in the recovery or the viability of the virus. The coupons (commercial biologic indicators) were purchased from Mesa Labs, France. The surface of the coupons has a hydrophilic treatment, which allows good distribution of the viral suspension over the entire surface of the coupon. Thus, each particle was exposed uniformly to the same conditions, avoiding any bias due to a thick deposit.

Dirty surfaces were simulated by adding 1% BSA and 1% yeast extract, as described in the NF EN 14675 standard (20).

**FIG 6:**
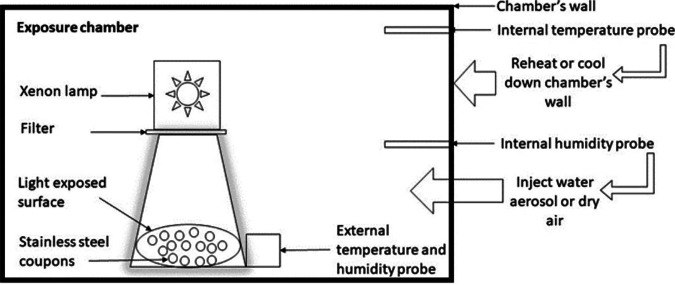
Schematic of the exposure chamber and its regulation system. An environmentally controlled chamber with a xenon light, which simulated sunlight, was used. In small stainless-steel coupons, SARS-CoV-2 was deposited and then dried at room temperature. Then, the coupons were put near the xenon lamp with different solar illumination parameters. The temperature inside the chamber was maintained at 20°C (± 2°C) by cooling or heating the wall of the chamber, depending on the measure. The relative humidity was adjusted to 50% (± 5%) by injecting dry air or by generating water aerosol, depending on the measure. In addition, an external probe was placed at the level of the coupon exhibition area, near the sunlight simulator, in order to assess these parameters locally.

The exposure to different climatic conditions was carried out in a chamber (shown in Fig. 6). The temperature (from 10°C to 45°C), relative humidity (from 10% to 80%), and solar illumination (from 10 klx to 56 klx) parameters can be regulated inside the chamber. The temperature and relative humidity parameters were continuously monitored in the chamber. In addition, an external probe was placed at the level of the coupon exhibition area, near the sunlight simulator, in order to locally assess these parameters.

The xenon lamp produces UV radiation from 290 to 400 nm (UVA and UVB). This spectrum includes a large part of solar UV radiation that naturally reaches the earth (from 280 nm to 400 nm). The illuminance can be adjusted between 10,000 lx, representing a covered sky in autumn in France, and 56,000 lx, representing a slightly cloudy sky in summer in France, using filters and a power controller. In comparison, solar illumination of 100,000 lx corresponds to illumination measured in June for a cloudless sky at this latitude.

All tests were carried out at a relative humidity of 50% (±5%) (value representative of summer conditions). By sampling at regular time intervals, kinetics analysis was performed for each condition tested. For each kinetic point, three coupons were taken. Each environmental condition tested was evaluated on three independent experiments.

A volume of 10 μl of viral suspension at 3.43 × 10^6^ TCID_50_/ml was deposited on each sterile coupon and dried in a safety biological cabinet for 45 min at room temperature. Then, the coupons were transferred to the chamber and exposed at different conditions.

For each experiment, time zero (T0) represents the concentration of infectious viral particles recovered in the coupons following 45 min of drying. Each point, including T0, was performed in triplicate. After exposure to climatic conditions, the coupons were collected and transferred to a tube containing 2 ml DMEM medium with GlutaMax supplemented with 2% heat-inactivated FBS and 1× antibiotic-antimycotic solution. After a 2-min vortex, the titer of each suspension was determined as described above.

### Statistical analysis.

We quantified the decay rate of SARS-CoV-2 under the different conditions using a Bayesian approach similar to the one described in Gamble et al. (44). We modeled well infections as a Poisson single-hit process to relate virus titer to positivity of well infection (45). Then, we estimated the decay rates of viable virus titer under environmental conditions using a Bayesian regression model. This modeling approach allowed us to account for differences in initial virus titers across samples, as well as other sources of experimental noise.

The model estimates the range of possible values for the viral decay rates and half-lives under various conditions, given our observed data. In addition, the Bayesian strategy gives us an estimate of the overall uncertainty (46).

Our prior on mean initial virus titers was based on the titer estimations obtained by the Reed-Muench method. Concerning log virus half-lives, we placed a weakly informative prior consistent with observations. The complete model is detailed in Text S1 in the supplemental material.

We estimated virus titers and model parameters by drawing posterior samples using pymc3 (47) which implements a No-U-Turn sampler algorithm (48). Existence of effects of luminosity, temperature, and/or cleanness of surfaces were evaluated based on probabilities of direction in order to give a Bayesian equivalent of *P* values (49).
