# Supplementary material for: Inactivation of SARS-CoV-2 by Simulated Sunlight on Contaminated Surfaces
Source: Microbiol Spectr. 2021 Jul 21;9(1):10.1128/spectrum.00333-21. doi: 10.1128/spectrum.00333-21 (PMC8552605; doi:10.1128/spectrum.00333-21)
Supplement: SUPPLEMENTAL FILE 1 — Supplemental material. Download SPECTRUM00333-21_Supp_1_seq1.pdf, PDF file, 0.4 MB. [file spectrum00333-21_supp_1_seq1.pdf]

Supplemental material

Table S1:

**Position of SNP with Wuhan strain as reference**	**Nucleotide on USA/WA-1 strain**	**Nucleotide on IDF-0372/2020**	**Protein impacted**	**Amino acid mutation**
8782 (C)	T	C	ORF 1 a/b	Synonymous
18060 (C)	T	C	ORF 1 a/b	Synonymous
22661 (G)	G	T	Spike	V367F
26144 (G)	G	T	ORF3	G251V
28144 (T)	C	T	ORF8	L84S

Comparison of Wuhan-HU-1 (GenBank: MN899049.3), BetaCoV/France/IDF-0372/2020 (GISAID: EPI_ISL_406596) and USA-WA1/2020 (GenBank: MT576563.1) genomes after alignment using progressive MAUVE aligner (1). The reference used for position of mutations is the strain Wuhan-HU-1.

Additional information S2: Bayesian estimation Model

In the following description, the symbol ~ denotes that a random variable is distributed according to the given distribution.

We denote $c_{i,j}(t)$ the titer for sample j under condition i measured at time t (minutes) in $\log_{10}$ $TCID_{50}$.

We assume an exponential decay of virus over time at a rate k_i (ki defined by $v_{i,j}(t) = v_{o,i}e^{-k_i*t}$, $v_{i,j}$ being the concentration in $TCID_{50}$) which depends on experimental condition i. It follows that the experimental titer in $\log_{10}$ $TCID_{50}$ is given by:

$$c_{i,j}(t) = c_{i,j}(0) - \beta_i * t$$

where $\beta_i = {}^{k_i}/_{\ln(10)}$.

We model the individual initial titers as normally distributed around the mean $\log_{10}$ titer for an experiment with a standard deviation compatible with observations:

$c_{i,j}(0) \sim Normal\left(\overline{c_\iota(0)}, \sigma_i\right)$, where $\overline{c_\iota(0)}$ and σ_i are respectively the mean and standard deviation of experimental initial titers evaluated with Reed and Muench method under condition i.

We used a Poisson single-hit model for positive and negative wells (2). The number of virions that will successfully infect cells within a given well, $n_{i,j}$, is therefore Poisson distributed:

$$n_{i,j} \sim Poisson(ln(2) * 10^{c_{i,j}(t)})$$

The mean of this distribution is determined by the fact that, according to the definition of $TCID_{50}$, we want the probability of having a positive well when $c_{i,j}(t) = 0$ (corresponding to an actual concentration of 1 $TCID_{50}$) to be equal to 0.5.

Let $W_{i,j,d,k,t}$ be a binary variable equal to 1 if the kth well at dilution factor d (d expressed in $\log_{10}$) for sample j under condition i at time t is positive and 0 otherwise.

According to previous models, we can assess that the probability of observing $W_{i,j,d,k,t}=1$ (a positive well) given titer $c_{i,j}(t)$ corresponds to the probability that our Poisson random variable is

greater than 0 with an expected concentration of $10^{c_{i,j}(t)-d}$. That gives the following formula for $P(W_{i,j,d,k,t} \mid c_{i,j}(t))$:

$$P(W_{i,j,d,k,t} \mid c_{i,j}(t)) = 1 - exp(-ln(2) * 10^{c_{i,j}(t)-d})$$

In accordance to these models, which are inspired from Gamble *et al.*(3), we can model the number of positive wells for given sample, condition and dilution with a Binomial probability distribution with parameters $p = 1 - exp(-ln(2) * 10^{c_{i,j}(t)-d})$ and n=6 (number of wells). The Binomial distribution is commonly used to describe the number of success for n independent draws of a binary event with a probability of success p. It is well adapted to our context. The number of positive wells is our observed data.

We placed a normal prior on the logarithm of half-lives ln(hi) ($h_i = ln(2)/\beta_i$) with a large standard deviation to be as uninformative as possible. Mean of the normal prior is egal to ln(30 minutes) for conditions in darkness, ln(5 minutes) for conditions with 10 klux luminosity and ln(2 minutes) for conditions with 56 klux. This arbitrary mean associated with a large standard deviation is a way to reflect slightly our assumptions on half-lives without constraining the Bayesian inference.

This modelling allowed us to infer virus titers, decay rates and half-lives under each conditions. Inferred titers were consistent with the ones evaluated by the Reed and Muench method.

References

1. Darling ACE, Mau B, Blattner FR, Perna NT. 2004. Mauve: multiple alignment of conserved genomic sequence with rearrangements. Genome Res 14:1394–1403.

2. Myers LE, McQuay LJ, Hollinger FB. 1994. Dilution assay statistics. J Clin Microbiol 32:732–739.

3. Gamble A, Fischer RJ, Morris DH, Yinda KC, Munster VJ, Lloyd-Smith JO. 2020. Heat-treated virus inactivation rate depends strongly on treatment procedure. BioRxiv Prepr Serv Biol https://doi.org/10.1101/2020.08.10.242206.
